# Effect of a Patient Engagement, Education, and Restructuring of Cognitions (PEERC) approach on conservative care in rotator cuff related shoulder pain treatment: a randomized control trial

**DOI:** 10.1186/s12891-023-07044-y

**Published:** 2023-12-01

**Authors:** Heather Myers, Francis J. Keefe, Steven Z. George, June Kennedy, Ashley Davis Lake, Corina Martinez, Chad E. Cook

**Affiliations:** 1 Urbaniak Sports Science Institute, Department of Rehabilitation Services, Duke University Health System, 3475 Erwin Rd, Durham, NC 27705 USA; 2 Department of Psychiatry and Behavioral Sciences; Psychology and Neuroscience, Medicine, and Anesthesiology, Duke University School of Medicine, 2200 W Main St, Suite 340, Durham, NC 27705 USA; 3 Department of Orthopaedic Surgery, Duke University School of Medicine, 200 Morris Street, Durham, NC 27701 USA; 4 Department of Orthopaedics, Duke University School of Medicine, 311 Trent Drive, Durham, NC 27705 USA

**Keywords:** Rotator cuff, Shoulder, Cognitive behavioral therapy, Expectations, Patient reported outcome measures

## Abstract

**Background:**

Despite similar outcomes for surgery and physical therapy (PT), the number of surgeries to treat rotator cuff related shoulder pain (RCRSP) is increasing. Interventions designed to enhance treatment expectations for PT have been shown to improve patient expectations, but no studies have explored whether such interventions influence patient reports of having had surgery, or being scheduled for surgery. The purpose of this randomized clinical trial was to examine the effect of a cognitive behavioral intervention aimed at changing expectations for PT on patient-report of having had or being scheduled for surgery and on the outcomes of PT.

**Methods:**

The Patient Engagement, Education, and Restructuring of Cognitions (PEERC) intervention, was designed to change expectations regarding PT. PEERC was evaluated in a randomized, pragmatic “add-on” trial in by randomizing patients with RCRSP to receive either PT intervention alone (PT) or PT + PEERC. Fifty-four (54) individuals, recruited from an outpatient hospital-based orthopedic clinic, were enrolled in the trial (25 randomized to PT, 29 randomized to PT + PEERC). Outcomes assessed at enrollment, 6 weeks, discharge, and six months after discharge included the patient report of having had surgery, or being scheduled for surgery (primary) and satisfaction with PT outcome, pain, and function (secondary outcomes).

**Results:**

The average age of the 54 participants was 51.81; SD = 12.54, and 63% were female. Chronicity of shoulder pain averaged 174.61 days; SD = 179.58. Study results showed that at the time of six months follow up, three (12%) of the participants in the PT alone group and one (3.4%) in the PT + PEERC group reported have had surgery or being scheduled for surgery (*p* = .32). There were no significant differences between groups on measures of satisfaction with the outcome of PT (*p* = .08), pain (*p* = .58) or function (*p* = .82).

**Conclusions:**

In patients with RCRSP, PT plus the cognitive behavioral intervention aimed at changing expectations for PT provided no additional benefit compared to PT alone with regard to patient report of having had surgery, or being scheduled to have surgery, patient reported treatment satisfaction with the outcome of PT, or improvements in pain, or function.

**Trial registration:**

The trial is registered on ClinicalTrials.gov: NCT 03353272 (27/11/2017).

## Background

Rotator cuff related shoulder pain (RCRSP) over-arching term that encompasses a spectrum of shoulder conditions including; subacromial pain (impingement) syndrome, rotator cuff tendinopathy, and symptomatic partial and full thickness rotator cuff tears [1]. This condition reflects 50%–85% of diagnoses for shoulder pain [1]. The exact prevalence of RCRSP is unknown, since most studies involve only rotator cuff tears, impingement syndrome only, or tendinopathy only or report values for selected age groups only. In trials involving rotator cuff related shoulder pain (RCRSP), conservative interventions such as exercise-based approaches have yielded similar outcomes with surgery [2–6]. However, despite the greater risk of harms, higher costs, and a high percentage of re-tears associated with a these procedures, the number of surgeries for all forms of RCRSP pain continues to increase [7–9]. For these patients, pre-treatment expectations of the success of surgical and/or conservative approaches are strongly associated with post-treatment outcomes [10–13]. The shoulder is not unique in these associations as patient expectations are understood to influence treatment outcomes for cervical, lumbar and lower extremity disorders [14–17].

Patient expectations are beliefs or attitudes that include pre-treatment thoughts and beliefs regarding the need for, timing, and intensity of specific treatment methods. Brief interventions designed to influence treatment expectations for physical therapy resulted in slight improvements in expectations, kinesiophobia and perceived disability [18, 19]. To date, no studies have explored whether adding cognitive behavioral intervention to change expectations for PT can influence patient reports of having shoulder surgery, or being scheduled for shoulder surgery. We posit that previous approaches to change patient expectations have had only modest effects because they do not include theory-based treatment techniques (e.g. CBT techniques) known to influence patient beliefs.

Our study purpose was to test an innovative intervention to alter expectations about physical therapy that is informed by principles of cognitive-behavioral theory: Patient Engagement, Education, and Restructuring of Cognitions (PEERC). The cognitive-behavioral therapy (CBT) treatment techniques that form the core of our PEERC intervention are patient-centered and are designed not only to alter expectations about PT but also decisions to pursue surgical treatment. The primary aim of this randomized clinical trial was to examine the effect of PEERC on the patient report of having had shoulder surgery, or being scheduled for surgery (primary outcome). Our secondary aims were to evaluate the impact of PEERC on expectations of treatment outcome during the course of PT, satisfaction of outcome of PT, pain and function (secondary outcomes).

## Methods

This randomized controlled trial was approved by the institutional review board of Duke University Health System and was registered with ClinicalTrials.gov (NCT03353272) (27/11/2017). The full trial protocol was previously published in *BMC Musculoskeletal Disorders* [20].

### Trial design

**Fig. 1:**
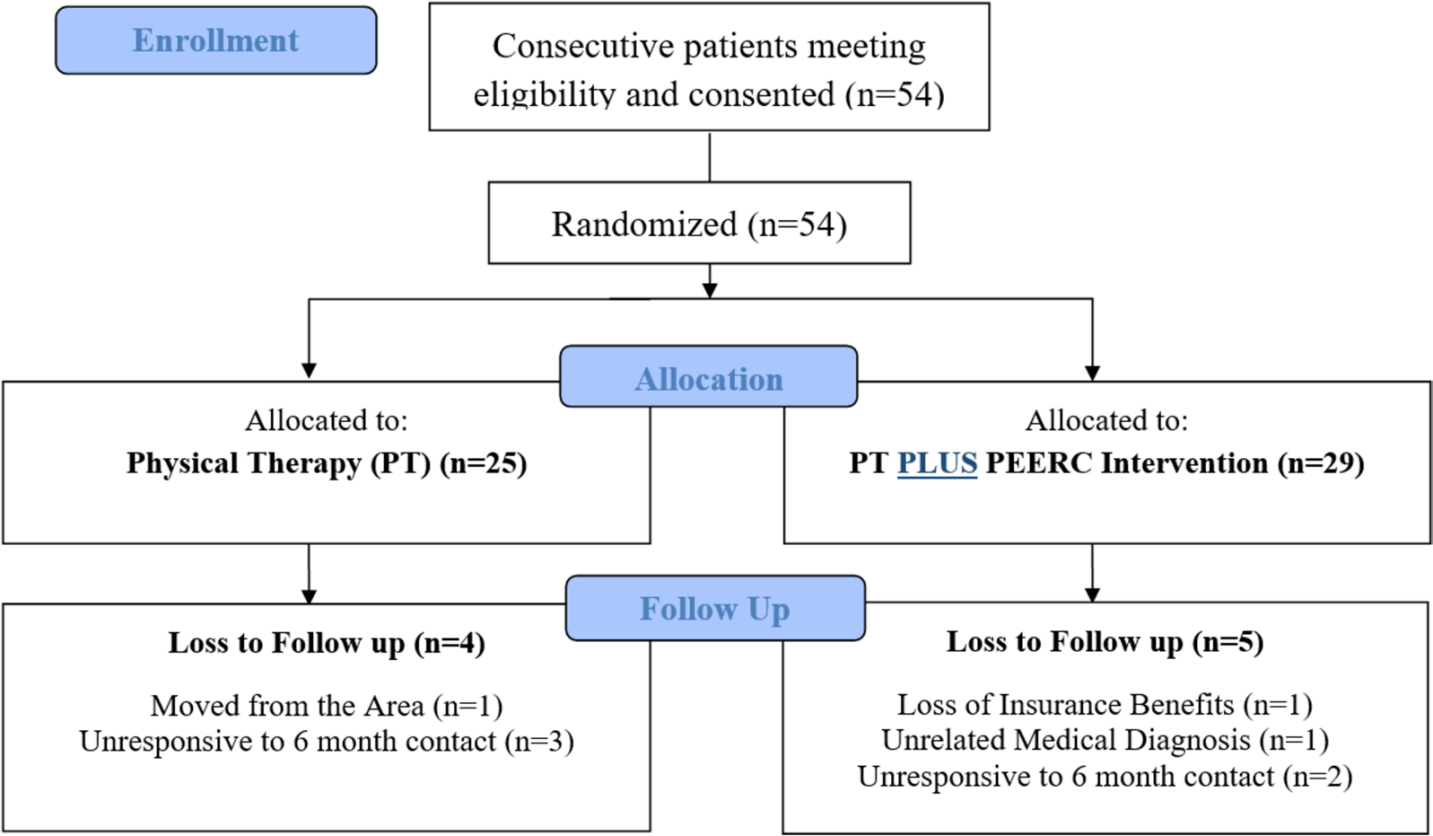
Consort flow diagram for enrollment, allocation, and follow up

Consented participants were randomized to receive either (1) an impairment-based physical therapy (PT) or (2) PT + PEERC group (Fig. 1). In this study, the impairment-based physical therapy only group served as the control. Participants were blinded to the study purpose of improving expectations of PT and the primary outcome (patient report of having had surgery, or being scheduled for surgery) by communicating that the investigators wished to improve the patient experience through additional education and interaction. Both groups received a dedicated musculoskeletal impairment-based, physical therapy approach that was pragmatic, but involved an established, three step phased approach supported by Kuhn [21], Garrison [22], and Stevenson [23]. In this approach, physical impairments identified on examination are then targeted with exercise to facilitate mobility, strength, and proper movement patterns. The phased approach allows patient-centered care that is unique to the needs of the patient and his/her progress, but reduces the variability of care that is common in physical therapy settings. Myers et al. outlines the staging criteria, goals, and sample exercises of the three phases used in this protocol [20]. To enhance treatment fidelity, the study therapists underwent a formal training program and used a treatment manual to guide their sessions. Adherence to the impairment-based treatment intervention was monitored via checklist for treatment fidelity by non-treating study personnel. Participant retention was promoted through contact between the physical therapist and the patient along with participant honorarium provided at the initial physical therapy evaluation and at the conclusion of ten weeks of active participation.

### Participants

Consecutive patients between 2018 and 2022 with RCRSP who were referred to Urbaniak Sports Sciences Institute by primary care physicians, orthopaedic surgeons, and physician assistants for physical therapy were recruited for the trial. Study activities were not initiated until after the patient provided written consent. The institutional review board of Duke University approved the study.

Inclusion criteria for this protocol included: ages 18 to 70; a mobile or land-line telephone; the ability to read, write, and speak English; and an RCRSP diagnosis inclusive of both acute and chronic cases. We excluded patients who had received, or were scheduled for, a surgical intervention for their shoulder condition, demonstrated any evidence of cervicogenic pain and/or radiculopathy from cervical origin, or who demonstrated symptoms consistent with thoracic outlet syndrome; all of which were identified during the clinical examinations by the attending physician and physical therapist. We also excluded individuals who were undergoing treatment for a serious psychological disorder (e.g., severe depression, psychosis).

### Randomization

Consented participants were randomized to receive either 1) PT alone or 2) PT + PEERC at the time of enrollment prior to PT evaluation. Consecutively numbered, sealed, opaque envelopes containing group allocation were prepared by a researcher with no other involvement in the study. Condition allocation involved randomization within random permuted blocks using the random number function in Excel and was stratified according to treating therapist so that all physical therapists will deliver approximately equal numbers of patients in both conditions to control for therapist variation.

### PEERC intervention

Patients in the PT + PEERC group received the PT intervention describe above *plus* a telephone-based intervention (designed by the authors), to challenge and change underlying thoughts, beliefs, and attitudes related to treatment expectations regarding PT care. PEERC, based on cognitive behavioral principles, was delivered by specifically trained physical therapists who conducted six 30-min telephone sessions with participants over a six-week period beginning the week after the initial evaluation. Treatment techniques used in PEERC were drawn from CBT to address issues related to thought distortions and irrational beliefs common in patients who have RCRSP. These techniques, are detailed in Myers et al. [20].

### Outcomes

Outcomes of interest were collected by study personnel at the time of consent, after 6 weeks of intervention, and at 6 months following discharge. Our primary outcome measure was patient report of having had surgery, or being scheduled for surgery, which was captured using a telephone survey at 6 months after discharge from impairment-based PT care. We selected a question framed around the patient’s choice of treatment approaches, and included the question: “Have you had surgery or are you scheduled for surgery for the shoulder problem that you were treated for in physical therapy?” A positive response was coded if the patient reported had already received surgery for their shoulder or was scheduled for surgery. We elected to use this question, because it allowed us to measure if patients had or were scheduled to have surgery in or outside of the study institution—in which case, a scheduled procedure would not be documented in the medical record.

Secondary outcome measures included changes in expectations while participating in PT and satisfaction with the PT outcome, as well as pain and function. Expectations and satisfaction with PT outcome were measured with the Musculoskeletal Outcome Data Evaluation Management System Expectations and Satisfaction Surveys (MODEMS-E and MODEMS-S) questionnaires respectively. The MODEMS [24–26] is a set of musculoskeletal assessment instruments created by the American Academy of Orthopaedic Surgeons. The MODEMS-E is a six-item instrument designed to capture patient expectations across a wide range of musculoskeletal conditions. The instrument is a Likert-based scoring tool with a mean score of 5 out of 5 (indicating high expectations of positive outcomes) and a mean score of 1 out of 5 (indicating very poor expectations of positive outcomes) [24]. The MODEMS–S consists of five similar stated questions from the MODEMS-E, but the questions are written to assess whether one’s expectations were met, indicating satisfaction with the outcome of PT. The MODEMS-S instrument is also a Likert-based scoring tool with a mean score of 1 out of 5 (indicating expectations were met) and a mean score of 5 out of 5 (indicating expectations were not met) [24]. Myers et al. details further description and the psychometric properties of each patient reported outcome included in the study [20].

Additional secondary outcomes included: shoulder pain intensity assessed through the Shoulder Pain and Disability Index (SPADI) [27–30] and the Numeric Pain Rating Scale (NPRS) [31–33], shoulder function measured with the SPADI, and Tegner Activity Scale (TEGNER) [34].

At baseline, we collected information on age, sex, marital status, education level, work status, prior episodes of PT (for current or different problem), if an injection was received, the Optimal Screening for Prediction of Referral and Outcome (OSPRO-ROS), and the Pain Catastrophizing Scale (PCS), both to describe participant presentation and to identify any post randomization difference between groups. To further describe and characterize participants after the course of treatment, we reported the Single Assessment Numeric Evaluation (SANE) [35–37], Global Rating of Change (GRoC) [38–42], number of PT visits, reason for discharge, and the patient’s overall experience with PT rated as excellent, good, fair, or poor.

### Sample size

We powered the study for proportional between-group differences in patient report of having had surgery, or being scheduled for surgery at a 6-month follow-up. Using projections from previous data, and assuming offset inequity between two independent conditions; we modelled power on several assumptions previously described in the protocol paper [20]. Our projected sample size was for 94 participants and, since we planned to employed intention to treat in the primary analysis, we did not account for dropouts in this projection.

### Statistical analysis

As previously described in the protocol paper, we evaluated descriptive statistics of the two conditions using appropriate parametric and nonparametric tests for differences, depending on the data (continuous or frequency based). For our primary outcome (patient report of having had surgery, or being scheduled for surgery), we measured condition differences in proportions between the PT only shoulder treatment and the PT + PEERC, using a chi-square analysis (or Fisher Exact). For our secondary outcomes, we used linear mixed effects modelling to compare follow up expectations and satisfaction of PT outcome (MODEMS) between the two conditions, as well as total SPADI scores, pain (SPADI), function (SPADI, TEGNER, GRoC, and SANE), and total visits. Repeated measures linear mixed effects modelling was used for all SPADI measures, pain, and GRoC scores. Pain intensity measures were evaluated using a negative binomial Poisson, which accounts for count variables with significant skew. A chi square analysis was used to calculated between group differences in patient experience ratings and discharge profile.

Effect size measures were calculated using Cohen’s d for continuous measures and an effect size index (w) for chi-square tests. We also evaluated partial eta squared (η^2^) for our repeated measures analyses. For Cohen’s *d*, the effect was considered trivial is *d* < 0.2, small if *d* = 0.2, medium if the *d* = 0.5, and large if the *d* = 0.8. For the effect size index *w,* the effect was considered trivial if *w* < 0.10, small if *w* = 0.10, medium if *w* = 0.30, and large if *w* = 0.50. For the partial eta squared values, the effect was trivial if η^2^ < 0.01, small if η^2^ = 0.01, medium if η^2^ = 0.06, and large if η^2^ = 0.14. All analyses were intention to treat and for each.

## Results

Fifty-four (54) individuals were enrolled in the trial. The average age was 51.81; SD = 12.54, and a majority were female (63%). The mean length of chronicity (days from symptom onset to physical therapy) visit was 174.61 days (SD = 179.58) and 20% had received physical therapy for a similar problem in the past. At baseline, the mean PCS score was 8.98; SD = 8.11, SPADI (total score) was 35.48; SD = 2.13, and MODEMS-E was 4.36; SD = 0.68. At baseline and prior to randomization, 70% of individuals either disagreed or strongly disagreed with the statement that they were interested in having surgery for their current condition. Table 1 includes additional baseline descriptive characteristics for both treatment groups. No obvious post-randomization differences were noted. Four participants in the PT group and 5 in the PT + PEERC group did not complete the study (Fig. 1).

**Table 1:** Baseline comparisons between physical therapy and PEERC versus physical therapy only

Variable	All subjects (*N* = 54) Mean (SD) / Number (%)	Physical Therapy and PEERC *N* = 29 Mean (SD) / Number (%)	Physical Therapy Only *N* = 25 Mean (SD) / Number (%)
Age	51.81 (12.54)	54.21 (13.05)	49.04 (11.56)
Sex	34 = Female (63%)	19 = Female (66%)	15 = Female (60%)
Educational Status	8 = High School (15%) 18 = College (33%) 9 = Graduate School-Doctorate (17%) 19 = Graduate School-Masters (35%)	7 = High School (24%) 9 = College (31%) 2 = Graduate School-Doctorate (7%) 11 = Graduate School-Masters (38%)	1 = High School (4%) 9 = College (35%) 7 = Graduate School-Doctorate (28%) 8 = Graduate School-Masters (31%)
Marital Status	44 = Married (81%) 3 = Divorced (56%) 6 = Single (11%) 1 = Widowed (2%)	24 = Married (83%) 2 = Divorced (7%) 3 = Single (10%) 0 = Widowed (0%)	20 = Married (80%) 1 = Divorced (4%) 3 = Single (12%) 1 = Widowed (4%)
Work Status	41 = Full time (76%) 5 = Part time (93%) 8 = Retired (15%)	19 = Full time (65%) 4 = Part time (14%) 6 = Retired (21%)	22 = Full time (88%) 1 = Part time (4%) 2 = Retired (8%)
Received Injection for Shoulder	26 = Yes (48%)	15 = Yes (52%)	11 = Yes (44%)
Onset to PT visit (days)	174.61 (179.58)	167.7 (181.53)	170.2 (173.31)
Received Physical Therapy Before (for a different problem)	27 = Yes (50%)	18 = Yes (62%)	9 = Yes (35%)
Received Physical Therapy Before for Current Problem	11 = Yes (20%)	5 = Yes (17%)	6 = Yes (24%)
Interested in Receiving Surgery for Current Problem	1 = Strongly agree (2%) 15 = Neither agree or disagree (28%) 11 = Disagree (20%) 27 = Strongly disagree (50%)	1 = Strongly agree (3%) 10 = Neither agree or disagree (35%) 4 = Disagree (14%) 14 = Strongly disagree (48%)	0 = Strongly agree (0%) 5 = Neither agree or disagree (20%) 7 = Disagree (28%) 13 = Strongly disagree 52%)
MODEMS-E score (1 to 5, 5 being highest)	4.36 (0.68)	4.34 (0.59)	4.36 (0.79)
OSPRO ROS (0 to 10)	2.22 (1.57)	2.28 (1.64)	2.16 (1.52)
Pain Score (0 to 10)	4.83 (0.68)	4.37 (2.39)	5.36 (7.83)
SPADI total score (0 to 100)	35.48 (21.31)	35.81 (23.33)	35.10 (19.16)
SPADI Pain (0–100)	47.55 (22.21)	49.02 (25.43)	45.84 (18.16)
SPADI Disability (0–100)	28.25 (23.92)	27.77 (24.62)	28.82 (23.58)
Pain Catastrophizing Scale (0 to 52)	8.98 (8.11)	7.89 (6.73)	10.24 (9.44)
PCS-Rumination	3.03 (3.19)	2.65 (2.90)	3.52 (3.56)
PCS-Magnification	2.40 (2.23)	2.06 (1.71)	2.84 (2.71)
PCS-Helplessness	3.38 (3.78)	2.72 (2.87)	4.08 (2.87)
Tegner Prior (0 to 10)	4.47 (1.70)	4.32 (1.59)	4.64 (1.84)
Tegner Current (0 to 10)	3.39 (1.46)	3.22 (1.33)	3.60 (1.61)

### Primary outcome

At six months (Table 2), one of the 29 (3.4%) in the PT + PEERC group and three (12%) of the PT only group reported having had surgery, or being scheduled for surgery, which was not different between groups (*p* = 0.32; *w* = 0.09).

**Table 2:** Surgery rate, SANE, and patient experience rating at six months

Variable	Physical Therapy and PEERC *N* = 29 Mean (SD) / Number (%)	Physical Therapy Only *N* = 25 Mean (SD) / Number (%)	Standardized Mean Difference (SE)	Effect Size (*d* and *w*)	*P* value
Patient report of having had surgery, or being scheduled for surgery	1 (3.7%)	3 (12%)	NA	*w* = 0.09 (trivial)	.46
SANE (0 to 100)	90.42 (5.35)	89.42 (13.57)	1.00 (2.73)	*d* = 0.09 (trivial)	.71
Patient Experience Rating	12 = Excellent (41.3%)	11-Excellent (44.0%)	NA	*w* = 0.03 (trivial)	.84

### Secondary outcomes

In our repeated linear mixed methods analyses, there were no significant differences between groups for pain (*p* = 0.67; η^2^ = 0.00), SPADI total score (*p* = 0.74; η^2^ = 0.00), SPADI pain score (*p* = 0.32; η^2^ = 0.02), SPADI disability (*p* = 0.97; η^2^ = 0.00), and the GRoC (*p* = 0.96; η^2^ = 0.00) (Table 3). At six weeks (Table 4), there were no between-group differences for MODEMS-E (*p* = 0.97; *d* = 0.02); MODEMS-E scores were similar to baseline measures in both groups. At discharge (Table 4), there were no between group differences in MODEMS-S (*p* = 0.08; *d* = 0.69), discharge profile (*p* = 0.37; *w* = 0.24), Tegner score (*p* = 0.89; *d* = 0.05), or total visits (*p* = 0.97; *d* = 0.01). At six months (Table 2), there were no between group differences for SANE (*p* = 0.71; *d* = 0.09) or patient experience ratings (*p* = 0.72; *w* = 0.03).

**Table 3:** Repeated measures comparisons of pain score, SPADI total score, SPADI pain, and SPADI disability scores

Variable	Physical Therapy and PEERC *N* = 29 Mean (SD)	Physical Therapy Only *N* = 25 Mean (SD)	Physical Therapy and PEERC *N* = 29 Mean (SD)	Physical Therapy Only *N* = 25 Mean (SD)	Effect Size (f)	*P* value
	Six Weeks		Discharge			
Pain Score (0 to 10)	1.65 (1.50)	1.43 (1.18)	1.59 (1.96)	1.65 (1.80)	η^2^ = 0.00 (trivial)	.67
SPADI total score (0 to 100)	15.99 (12.03)	15.37 (6.75)	7.48 (4.40)	9.79 (6.09)	η^2=^0.00 (trivial)	.74
SPADI Pain (0–100)	21.53 (13.01)	23.28 (10.81)	13.30 (8.48)	16.71 (10.18)	η^2=^0.02 (small)	.32
SPADI Disability (0–100)	12.48 (11.67)	11.01 (8.01)	3.61 (2.39)	6.10 (6.34)	η^2=^0.00 (trivial)	.97
Global Rating of Change (GRoC) (-7 to 7)	4.77 (1.93)	4.84 (1.89)	4.60 (1.54)	4.48 (2.30)	η^2=^0.00 (trivial)	.96

All analyses include baseline controls of the same variable with the exception of the GRoC

**Table 4:** Six-week and discharge comparisons between physical therapy PLUS PEERC versus physical therapy only

Variable	Physical Therapy and PEERC *N* = 29 Mean (SD) / Number (%)	Physical Therapy Only *N* = 25 Mean (SD) / Number (%)	Standardized Mean Difference (SE)	Effect Size (*d* and *w*)	*P* value
Six Week Outcomes					
MODEMS-E score (1 to 5; 5 is best)	4.36 (0.51)	4.35 (0.60)	-0.01 (0.15)	*d* = 0.02 (trivial)	.97
Discharge Outcomes					
Global Rating of Change (GRoC) (-7 to 7)	4.60 (1.54)	4.48 (2.30)	0.11 (.53)	*d* = 0.06 (trivial)	.83
Tegner Score	4.27 (1.16)	4.32 (0.93)	-0.5 (.33)	*d* = 0.05 (trivial)	.89
Discharge Profile	10 = Discharge by PT (35%) 13 = Self Discharge (45%) 1 = Surgery (3%) 5 = Other (17%)	13 = Discharge by PT (52%) 10 = Self Discharge (40%) 1 = Surgery (4%) 1 = Other (4%)	NA	*w* = 0.24 (small)	.37
SANE (0 to 100)	90.42 (5.35)	89.42 (13.57)	1.00 (2.73)	*d* = 0.09 (trivial)	.71
Total Visits	4.42 (3.22)	4.45 (2.94)	-0.03 (0.86)	*d* = 0.01 (trivial)	.97
MODEMS-S score (1 to 5; 1 is best)	3.62 (0.62)	3.13 (0.79)	0.48 (0.34)	*d* = 0.69 (medium)	.08

## Discussion

Our study examined the effect of PEERC, an intervention designed to improve expectations of PT, on patient report of having had surgery, or being scheduled for surgery (primary outcome). In this manner, PEERC is a novel form of psychologically informed physical therapy as it was not designed to directly address pain associated distress. Our secondary aim was to evaluate the impact of PEERC on expectations and satisfaction with PT outcome, pain, and function. We did not find differences between PT only and PT + PEERC in our primary outcome at six months follow up, nor did we identify between group differences for any of the secondary outcomes included in this trial.

There are several potential reasons we did not detect differences between PT and PT + PEERC. Firstly, similar to many studies performed in the 2020 to 2022 timeframe [43–45], enrollment was challenging, especially for studies that involved patients seeking elective surgeries. Because of a lengthy COVID lock-down, and continued inaccessibility to see patients “live”, we were unable to enroll the projected sample size estimate (*N* = 94) for our study. The lack of full enrollment suggests that our trial was likely underpowered. Further, our study was an “add on trial” (A versus A + B design), which generally requires a larger number of patients to see differences when analyzed [46]. Nonetheless, looking at the current findings and assuming consistent trends in the data for effect size (*w* = 0.09), post-hoc power analysis recommends we would have needed a sample size of 236 to show group differences for our primary outcome. In addition, with the exception of a medium effect size (*d* = 0.69) for the MODEM-S score and a small effect size (*w* = 0.24) for discharge profile (basis for discontinuation of PT); all other between group effect sizes were *trivial*. Taken together, the pattern of results obtained suggests that although we did not achieve the a priori determined sample size of 94, we would not have observed notable differences to what we found with our current sample size because a much larger sample would have been needed to detect between group differences. Second and related to the small sample size, the use of a binary endpoint was another limitation in that it limited our statistical power relative to if we had an endpoint on a continuous scale.

Third, we used an A versus A + B design to measure the effectiveness of the addition of the PEERC intervention. In theory, this design allows the investigators to understand better the true, isolated ‘effect’ of the “add-on” intervention [47]. Add-on designs are especially useful for testing of experimental interventions with mechanisms of action different from that of the established, effective treatment [48]. Traditional, physical impairment based physical therapy care for RCRSP (which both groups received) involves strengthening and range of motion exercises, as well as a home exercise program. Our trial incorporated best recommended physical therapy treatment practices and accordingly this may be another reason PT alone and PT + PEERC had similar rates for patient report of having had surgery, or being scheduled for surgery and all secondary outcomes. Compared to baseline values for pain, and the SPADI scores associated with pain, disability, and total scores, both groups markedly improved.

The hallmark of the PEERC intervention is the targeting of maladaptive cognitions and provision of compensatory pain management strategies for those with ongoing pain in order to improve patient expectations for treatment. Our pain catastrophizing (PCS) baseline measures were low for the PT arm (10.24; SD = 9.44) and even lower in the PT + PEERC arm (7.89; SD = 6.73). It has been suggested that pain catastrophizing scores of 30 or greater are considered clinically relevant level of catastrophizing in populations with chronic pain [49, 50]. Our inclusion criteria, which allowed both acute and chronic RCRSP, resulted in patients with a wide range of chronicity (0 to 725 weeks). The average SPADI total scores were very low in comparison to similarly reported populations [51–54], suggesting self-report of disability levels were not severe. Further, nearly every patient enrolled reported very favorable expectations (4.36; SD = 0.68 / 5.0) about their assigned conservative care, lessening the likelihood that the patient expectation modifications were necessary. At baseline, only three of our 54 enrollees (5.5%) had expectations scores of 3/5 or lower, which we originally projected would be necessary to optimize the PEERC effect. Up to 20% of individuals had received physical therapy before, which may have also influenced expectations.

Considered as a whole, the baseline characteristics of our study population suggest there may have been a “mismatch” between the patients we recruited and the goals of PEERC. That is, a majority of the study sample (70%) told us at baseline that they were not interested in having surgery and already had reasonably high expectations of PT care delivered by physical therapists. Simply stated, there is chance that the PEERC intervention is potentially beneficial, but the study was conducted with a population who is not as likely to need or benefit from it.

We noticed a number of intriguing issues during implementation of the PEERC health coaching. Although PEERC included six visits, over a six-week timeframe, with phone calls serving as the medium, it was clear that in multiple occasions, patients did not participate in the PEERC health visits at the level that we had hoped. Despite requests to dedicate time to the full session (as outlined in the daily fidelity sheets), there were several times in which PEERC calls occurred during inopportune times; 1) while the patient was driving a car, 2) attending or coaching their youth’s sporting events, 3) while at work, 4) while cooking dinner, or 5) during other activities in which they multi-tasked the cognitive behavioral strategies of the PEERC with other daily activities. There were multiple occasions where a lack of preparedness from the patient was evidenced in the PEERC homework activities. This may be related to our selection of a phone to interact with the PEERC group. Although the use of phone allows broader accessibility, in situations such as cognitive behavioral based approaches, where non-verbal cues relationship building between patient and provider are known to enhance treatment, video may be a better alternative [55]. We would certainly recommend this moving forward beyond this study along with strategies to oblige better compliance with homework preparedness.

### Limitations

In addition to the aforementioned sample size and composition issues, the following limitations are worth noting. Patients were participants referred for a physical therapy treatment program for RCRSP; consequently, there is a high risk of selection bias (e.g., the patients expected physical therapy to work and did not feel surgery was necessary), which may also be the reason for the high baseline expectations for treatment. In order to remedy this recruitment issue, future research should be designed to target recruitment to individuals that are most likely to have lower expectations of physical therapy treatment). Another participant related limitation to consider is that the collection of data did not allow for differentiation of sex and gender identity. Depending on the research question, this distinction may be important to make in future research of interventions like PEERC.

In this trial we intentionally used an A versus A + B design as it bested addressed our research question. However, this design could be considered a limitation by those interested in the isolated effects of PEERC. Therefore, future research could consider A versus B designs to address this design choice. PEERC was provided by two experienced, PhD-level physical therapists who had undergone formal external (e.g., educational certificate on pain management) and within-study (dedicated training sessions for the study) training. Nonetheless, both individuals were not psychologists and there is the chance that this influenced the overall quality of the interventions, particularly having the background, experience, and skills to optimize its effectiveness. Also, although a fidelity checklist and “daily coaching” sheets were used to maintain treatment fidelity, there is a risk that some therapist drift may have influenced the uniformity of the interventions. Another limitation is that the primary outcome was collected by self-report. Thus, patient reports of having had surgery or being scheduled for surgery were not verified via electronic medical record. This approach was determined to be acceptable for this trial because the trial team had ready access to this patient population and a manageable (*n* < 100) sample size was planned which allowed us to measure if patients had, or were scheduled to have, surgery inside or outside of the study institution. However, in future studies involving different clinical centers and larger sample sizes it would be good practice to have verification of this primary outcome via the medical record.

## Conclusions

A novel six-week cognitive behaviorally-based intervention to alter expectations for PT(PEERC) provided no additional benefit when used as a routine adjunct to conventional PT alone for patients with RCRSP. Although planned sample size estimates were not met, post-hoc power analyses suggest that a substantially larger sample size than projected, or substantially larger treatment effects than observed, would be necessary to show benefits of PEERC on our primary outcome (patient reports of having had surgery or being scheduled for surgery). Therefore, it is difficult to advocate for PEERC adding value to PT alone in the management of RCRSP for individuals matching the characteristics of the patients enrolled in this trial (i.e. with high expectations of physical therapy). Future work in patient populations that are screened for high levels of surgical interest, with higher levels of pain associated distress, and/or poor expectations with physical therapy would be necessary to fully evaluate the potential value of PEERC.

## Data Availability

The datasets used and/or analyzed during the current study are available from the corresponding author on reasonable request.

## Abbreviations

PEERC: Patient Engagement Education and Restructuring of Cognitions
CBT: Cognitive Behavioral Therapy
RCRSP: Rotator Cuff Related Shoulder Pain
MODEMS: Musculoskeletal Outcome Data Evaluation Management System
SPADI: Shoulder Pain and Dysfunction Index
OSPRO-ROS: Optimal Screening Prediction of Referral and Outcome
PCS: Pain Catastrophizing Scale
SANE: Single Assessment Numeric Evaluation
TEGNER: Tegner Activity Scale
NPRS: Numeric Pain Rating Scale
VAS: Visual Analog Scale
GRoC: Global Rate of Change
